# Temperature and Light-Quality-Dependent Regulation of Freezing Tolerance in Barley

**DOI:** 10.3390/plants9010083

**Published:** 2020-01-09

**Authors:** Mohamed Ahres, Krisztián Gierczik, Ákos Boldizsár, Pavel Vítámvás, Gábor Galiba

**Affiliations:** 1 Festetics Doctoral School, Georgikon Faculty, University of Pannonia, 8360 Keszthely, Hungary; ahres.mohamed@agrar.mta.hu; 2 Agricultural Institute, Centre for Agricultural Research, 2462 Martonvásár, Hungary; gierczik@gmail.com (K.G.); akos.boldizsar@gmail.com (Á.B.); 3 Department of Genetics and Plant Breeding, Crop Research Institute, 161 06 Prague 6, Czech Republic; vitamvas@vurv.cz

**Keywords:** barley, frost tolerance, *HvCBF14*, CBF regulon, LED lighting, far-red light, low temperature

## Abstract

It is established that, besides the cold, incident light also has a crucial role in the cold acclimation process. To elucidate the interaction between these two external hardening factors, barley plantlets were grown under different light conditions with low, normal, and high light intensities at 5 and 15 °C. The expression of the *HvCBF14* gene and two well-characterized members of the C-repeat binding factor (CBF)-regulon *HvCOR14b* and *HvDHN5* were studied. In general, the expression level of the studied genes was several fold higher at 5 °C than that at 15 °C independently of the applied light intensity or the spectra. The complementary far-red (FR) illumination induced the expression of *HvCBF14* and also its target gene *HvCOR14b* at both temperatures. However, this supplementation did not affect significantly the expression of *HvDHN5.* To test the physiological effects of these changes in environmental conditions, freezing tests were also performed. In all the cases, we found that the reduced R:FR ratio increased the frost tolerance of barley at every incident light intensity. These results show that the combined effects of cold, light intensity, and the modification of the R:FR light ratio can greatly influence the gene expression pattern of the plants, which can result in increased plant frost tolerance.

## 1. Introduction

Cold acclimation is the prerequisite of overwintering for different plant species growing at temperate climates in the northern hemisphere. In order to achieve the genetically determined full winter hardiness, plants have developed different strategies [1]. For example, entering the period of dormancy is an essential strategy for perennial plants to survive harmful freezing conditions during winter [2]. Concerning the economically most important grasses like the cereals, they must go through a lengthy cold acclimation period, which requires the orchestration of transcriptional, biochemical, and physiological changes [3]. In both cases, cold acclimation is associated with decreasing the photoperiod and cessation of growth [4,5]. Recently accumulated evidence has elucidated that the “master gene(s)” behind these well-orchestrated acclimation processes both in perennial and annual plant species are the C-repeat binding factors (CBFs). The CBF transcription factors belong to the dehydration-responsive element binding factors (DREB1) subfamily of the APETALA 2/ethylene-responsive element binding factor (AP2/ERF) protein family. They are able to bind to the C-repeat/dehydration responsive element (CRT/DRE) sequence motif in the promoter region of the regulated gene, which contains a conserved CCGAC sequence as a binding site for the DNA-binding domain of CBF proteins [6,7]. Their crucial role in the cereal cold hardening process was revealed by quantitative trait locus (QTL) mapping [8,9,10]. Quantitative genetic studies in barley, in diploid einkorn wheat (*Triticum monococcum* L.) and in hexaploid wheat (*Triticum aestivum* L.) have shown that a large number of phenotypic differences in frost tolerance and winter hardiness are explained by two QTLs: the *Fr-1* and *Fr-2* (frost resistance) loci. It became clear that the *Fr-1* locus on the 5A chromosome harbours the *VRN1 vernalization* gene, which regulates the transition of the vegetative shoot apical meristem to the reproductive phase; consequently, it just has a pleiotropic effect on frost tolerance [11,12,13]. On the other hand, at least 11 *CBF* genes have been mapped in the *Fr-2* locus within a small 0.7–0.8 centimorgan distance in diploid wheat and barley, clearly showing that the *CBF* genes are the best candidate genes for those QTLs [9,10,14,15,16,17]. Otherwise, the crucial role of CBFs to induce dormancy in perennial species became obvious when a full-length cDNA of a peach *CBF* gene (*PpCBF1*) was isolated by Wisniewski et al. [18], and its ectopic expression in apples resulted in induced dormancy and increased cold hardiness. *CBF14* is an important gene for winter survival in cereals, as demonstrated by increased frost tolerance in transgenic spring barley plants (*Hordeum vulgare* L. ‘Golden Promise’) expressing wheat *TaCBF14* at a non-acclimating temperature [19]. In addition, the variation in cold hardiness levels among barley genotypes is associated with *CBF14* allelic differences [20].

It is also well established that, in addition to low temperature, light also acts as an external signal and affects the expression level of the *CBF* genes [5]. It has been described that the photoperiod and the light quality are important regulators in the cold acclimation processes through the modulation of the CBF regulon [21,22,23,24]. By using artificial light conditions, it was observed that the effect of temperature and light treatments on *CBF* gene expression is additive [23]. To make the picture more complicated, it is also known that light intensity itself affects the degree of frost tolerance since low light intensity can mitigate the level of freezing tolerance. [25].

Genes affected by CBF transcription factors are collectively referred to as the CBF regulon [26]. The most important of these genes are the members of the cold regulated gene (CORs) family, also known as low-temperature induced (LTI), cold-inducible (KIN), and responsive to desiccation (RD) family [27] and the dehydrin (DHN) family. The COR genes primarily contribute to an increase in freezing tolerance until the DHN genes protect against the adverse effects of water loss caused by freezing [28,29,30,31]. The first direct evidence of this phenomenon was the discovery of the dehydration-responsive element (DRE), which contributes to the expression of both cold- and dehydration-induced genes, but has no effect on the expression of abscisic acid (ABA) regulated genes. [32]. It was also proved that in light-mediated signalling the CBFs play an important role in the transcriptional regulation of CORs [33].

From the COR gene family, *HvCOR14b* is the best characterized among the molecular genetic factors in the field of frost tolerance in cereals [34]. The expression level of this gene is mostly dependent on the cold sensitivity of the given plant. In frost-tolerant wheat genotypes, the accumulation of *TaCOR14b* starts even at 18/15 °C temperature until in frost-sensitive genotypes they are not expressed at all [3,29,35].

The *dehydrin 5* (*HvDHN5)* gene is the most cold-inducible dehydrin in barley and is an orthologue to the *Wcs120* gene in wheat [36,37]. In wheat, the *WCS120* gene can be used as a marker gene for frost tolerance [38]. In barley, the *HvDHN5* gene is also considered as a marker gene for the maximum acquired frost tolerance in barley; however, in contrast to the *WCS120* gene, the expression level of the *HvDHN5* gene is only a part of the whole system [39].

The photoperiod is also an influencing factor in the development of cold acclimation in barley [40]. In this experiment, we used a winter habit barley *Hordeum vulgare* spp. *vulgare* var. Nure. This genotype carries two photoperiod-sensitive (PPDH) alleles. One of them is the PPD-H1 (on chromosome 2H), which is insensitive (ppd-H1) for day length. The other one is the PPD-H2 (on chromosome 1H), which is the sensitive allele (Ppd-H2) [41]. Under intermediate photoperiods, these genes do not activate at all; thus, in this study, it was not necessary to study the PPD genes.

There are at least two important reasons to elucidate how variable light conditions and temperature affect the cold acclimation process of plants: one of them is climate change and how it affects plant cold acclimation. If we understand how the plants respond at a molecular level to temperature fluctuations, we might get clues for improvement the freezing tolerance under the predicted future climate [42]. The second reason is more practical: to improve winter hardiness, the breeders frequently use plant growth chambers to select for freezing tolerance. In the case of cereals, our whole plant frost testing program ran for 8 weeks, and if we used two selection temperatures (like −12 and −15 °C), we could only test 60 genotypes in one plant growth chamber [9,19,43,44,45]. So, it is rather expensive and time consuming. Still, there is a problem: if the freezing tolerance of the individual lines of the tested germ plasm just slightly differ (1 or 2 °C), the results will hardly be repeatable. In addition, the aging of the frequently used cool white fluorescent tubes results in the alteration of light intensity and also the spectrum of the emitted light.

To draw attention to the importance of the applied temperature, light intensity, and spectrum in the process of cold acclimation, we designed an experiment where LED light sources were used. Thus, our aim was to study the expression of the *HvCBF14* gene and two well-characterized members of the CBF-regulon, the *HvCOR14b* and the *HvDHN5* genes, under modulated light and temperature conditions. We also elucidated how light intensity, in parallel with the modified spectrum of incident white light, affects the freezing tolerance of the barley seedlings.

## 2. Results

### 2.1. The Expression Patterns of HvCBF14 and Its Target Gene HvCOR14b

**Figure 1:**
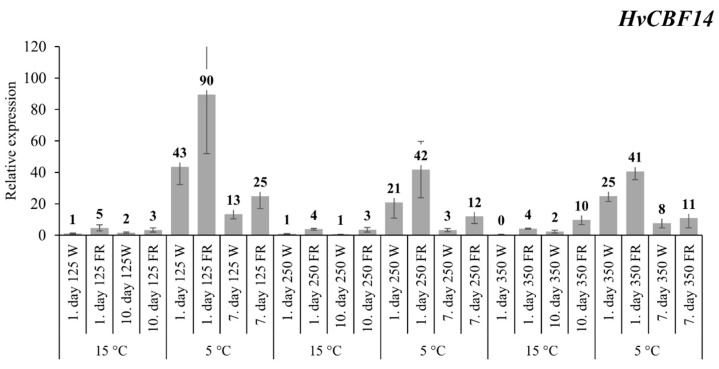
The expression pattern of the *HvCBF14* gene with white light and low R:FR illumination. The examined plantlets were grown under 12 h photoperiod where the samples were collected six to eight hours after the illumination began. The *X*-axis shows the phases of the treatments; Transcript levels were calculated with the ΔΔCt method. The data and error bars, which represent the standard deviation, originated from three biological replicates with three technical samples.

Using the ΔΔCt method, we evaluated the samples grown under low light intensity and white light at 15 °C and were considered as the controls. FR supplementation at low light intensity (125 photosynthetically active radiation (PAR)) caused a 5-fold increase in the *HvCBF14* transcript level at 15 °C on the first day of the experiment, but after ten days this difference faded to 3-fold (Figure 1). After one day at a low 5 °C temperature, there was a large increase in *HvCB14* gene expression (about 43-fold) even under white light illumination, but as a result of FR supplementation, this increase was even doubled. By the seventh day of the treatment, the expression levels had fallen, but the elevated pattern remained the same. At normal and high light intensity, 250 and 350 PAR respectively, similar gene expression patterns were obtained. The only important differences were the following: lower relative levels were achieved compared to the low-intensity results after cold exposure, but there were clear differences between the treated and untreated samples at all times.

**Figure 2:**
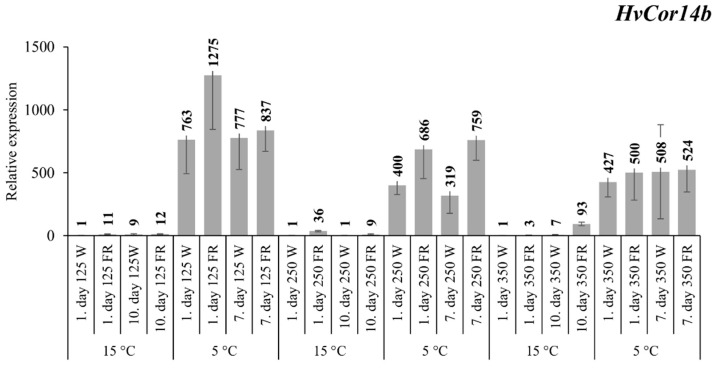
The expression pattern of the *HvCOR14*b gene with white light and low R:FR illumination. Conditions are the same as in Figure 1.

For *HvCOR14b*, the same procedure was used for evaluation (Figure 2). The results were similar to *HvCBF14′s* patterns, so it confirmed its expression values as well. At low light intensity (125 PAR) and as a result of FR supplementation, *HvCOR14b* expression increased 11-fold on the first day, and this high level of expression was maintained until the end of the ten-day acclimation period at 15 ℃. At low temperature, a staggering increase was measured. There was more than a 700-fold increase in the gene expression levels in the samples illuminated by white light, and still this value almost nearly doubled due to FR light supplementation after one day. However, the difference almost completely disappeared after the seven-day cold acclimation period. At normal (250 PAR) light intensity there were also clear differences in all cases, similarly to the *HvCBF14* transcription factor. At 15 ℃ the expression was increased 36-fold on the first day and 9-fold on the last day. At 5 ℃ a two-fold difference was measured between the light treatments in both cases, while the expression was significantly increased by the cold. At high light intensity, mRNA levels did not alter as a function of decreased R:FR ratio at 5 °C. There was no difference between the control and the FR treated samples; only the cold had an effect on the expression of this gene (Figure 2). However, at 15 °C FR supplementation caused a massive 93-fold increase in the transcript level after the ten-day treatment.

### 2.2. The Expression Levels of the HvDHN5 Gene

**Figure 3:**
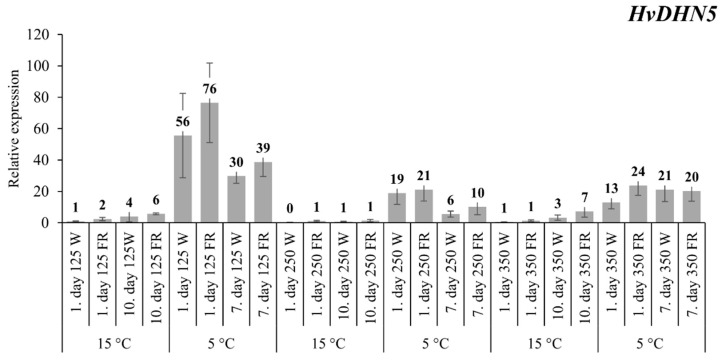
The expression pattern of the *HvDHN5* gene with white light and low R:FR illumination. Conditions are the same as in Figure 1.

At low 125 PAR incident light intensity, the transcript level of the *HvDHN5* gene doubled as a result of FR supplementation on the first day at 15 °C, but this difference diminished after 10 d of the treatment (Figure 3). This tendency was also evident at 5 °C. Based on the results from samples treated at higher light intensities, it can be generally stated that neither the temperature nor the light treatments had a significant effect on the gene expression pattern. Of course, the gene expression levels were permanently higher in the samples originating from the lower temperature (5 °C). 

### 2.3. The Results of the Relative Conductance Measurements

**Figure 4:**
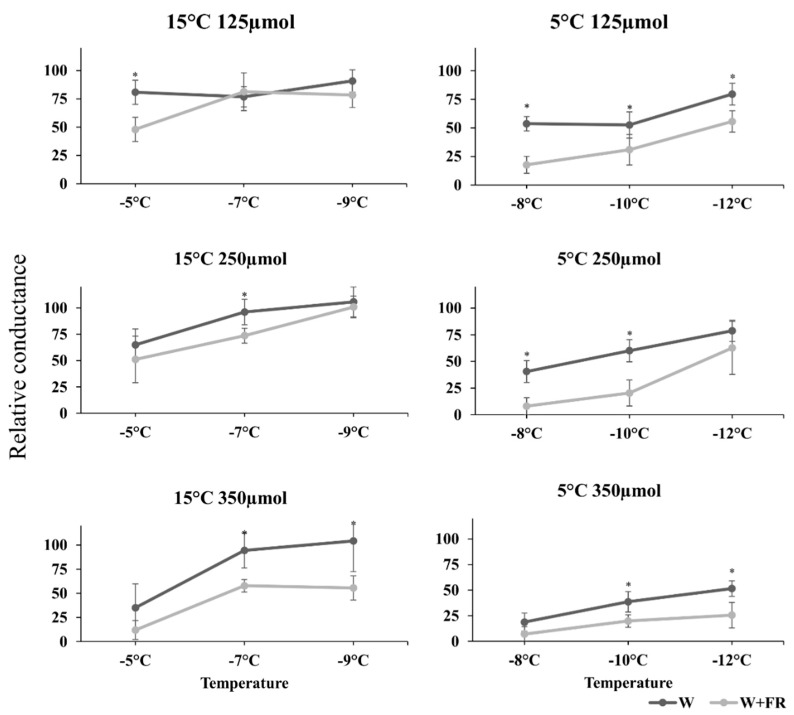
The effects of the supplementary far-red light treatments on freezing tolerance under various light intensities in barley plantlets. The *X*-axis shows the freezing temperatures while the *Y*-axis refers to the relative conductance values (percentage of the lethality). In each case, the samples were collected on the last day of the treatments. In 15 °C temperature tables it means the 10th day, while in the 5 °C temperature tables it was on the 7th day. The data and error bars, which represent the standard deviation, originated from five biological replicates.

To test the physiological effects of modulated illumination on the acquired freezing tolerance of barley seedlings, freezing tests were performed. The freezing tolerance was determined by relative conductance measurements of leaf segments frozen at different temperatures. The analysis was carried out after 7 and 10 d of treatment with FR-modulated white light using leaf samples originating from seedlings grown at 5 and 15 °C, respectively (Figure 4). The comparisons were made directly to white light against light with low R:FR ratio treated samples. In the case of plants grown at low (125 PAR) incident light intensity, at 15 °C a significant difference was observed, but only in samples that were frozen at −5 °C. The FR-treated plants were just around the LT50 (lethal temperature), while the control white light illuminated samples reached 75% lethality. In contrast, at 5 °C, which is low enough to induce cold acclimation, the plants exposed to modulated white light were more frost hardy. These plants reached the LT50 only after freezing at −12 °C, whereas in the case of the control, white light illuminated samples, the LT50 was reached at −8 °C. At increased light intensity (250 PAR), illumination by white light with low R:FR ratio lowered frost-induced cell membrane injury in leaves even to a higher extent. When the highest light intensity (350 PAR) was applied, the relative conductance of the control samples decreased considerably. But, even in this case, FR supplementation caused significantly more reduction (Figure 4). Similar to the samples illuminated by lower light intensities, the freezing tolerance of the plants kept at low temperature (5 °C) were considerably higher than that of their counterparts grown at 15 °C.

## 3. Discussion

### 3.1. The Expression of HvCBF14 and Its Target Genes HvCOR14b and HvDHN5

Previously, we reported that the expression level of the *HvCBF14* gene was increased at 15 °C, even at a noninductive 22 °C, as a result of the decreased R:FR ratio of the incident white light after one day, but this modification made no changes in its expression at a low 5 °C temperature [23]. In light of this present paper (Figure 1), we must revise that report. The alteration most likely was due to the difference in the used light sources. In 2016 we used Sylvania 215WF96T12 cool white linear fluorescent tubes instead of LED light sources. Supplementary FR (735 nm) was added by 3W high-power light-emitting diodes to produce low R:FR ratios in the spectrum. In fluorescent tubes, the spectrum of the light is not consistent because it contains several prominent blue peaks that may have interfered with *HvCBF14* expression (Novák et al. 2016; Figure S1). In cereals, there is no evidence for this theory, but in *Arabidopsis thaliana* the *COR27* and *COR28* genes were shown to be repressed by blue light, which negatively regulated *CBF* expression [46]. Considering the present experiment, it can be clearly seen that, under cold acclimation conditions, with a well-defined, clear spectrum, far-red light is even more stimulating and enhances the expression levels (Figure S1). Interestingly, the highest *HvCBF14* transcript levels were obtained with the lowest light intensity, suggesting that plants were under stress conditions (not optimal levels for photosynthesis) and, therefore, they were more responsive (Figure 1). We must also consider that, in a natural environment, a decreased R:FR ratio occurs at sunset and, during this twilight period, the light intensity is gradually decreasing. Consequently, it seems to be evident that not only the spectrum but also the changing light intensity can be an important, external signal affecting gene regulation [21,24,47,48]. In tomatoes, monochromatic red and far-red light was tested for inducing cold tolerance. They found that when using only red light, the relative electrolyte leakage level was much higher than in the case of far-red illumination. From that, the transcript level of *CBF1* was reduced by the radiance of monochromatic red light, while an increase was measured by using only far-red lighting, but only after cold treatment [49]. 

Our *HvCOR14b* expression results were similar to *CBF14* mRNA accumulation (Figure 1 and Figure 2). This result also proved that regulation of the *COR* genes is influenced not only by the cold but also controlled by light conditions, or at least *HvCOR14b* is definitely affected by the light. Kobayashi et al. (2004) demonstrated that a dark environment inhibits, while the light stimulates, the expression of several COR genes, including *COR14* in wheat. At 15 °C, there was an increase in the expression of this gene even under white light after ten days of light intensity modifications, which clearly correlate to Kobayashi’s and Vágújfalvi’s results in wheat [35,50]. In this case, there were also huge differences in each intensity resulting from far-red supplementation as well. Previously, in etiolated barley plants, the expression of *HvCOR14b* was also found to be determined by the light quality. Red and blue light were able to raise the transcript level of the gene, while far-red and green light did not affect it at all [51]. Our results showed that, in normal conditions, far-red light can be a very influential factor to its expression as well. At low (125 PAR) and normal (250 PAR) intensities, there was a significant change on the first day of FR treatment, whereas at high intensity, there was a massive 93-fold increase on the last day at 15 °C. In contrast, at low 5 °C temperatures, only small differences were detectable between the treated plants. It means that at high intensity with low temperatures there was a threshold in the induction of the gene, and it was not induced further by FR supplementation, unlike in other treatments. The biggest changes occurred at low intensities, such as in the case of *HvCBF14* expression. This well illustrates the effects of both intensity and quality of light on *HvCOR14b* expression.

The *HvDHN5* gene turned out to be much less responsive than the other two genes examined for the FR-modulated spectra (Figure 3). Only the effect of temperature on its transcript level was obvious. This result was expected because it had been presented that, under a controlled environment, the accumulation of *HvDHN5* was increased by the cold in many genotypes, and *HvDHN5* can be regarded as a marker gene for frost tolerance [39] in barley. Interestingly, *HvDHN5* expression was not significantly altered with elevated HvCBF14 levels. This may be related to the fact that the *HvDHN5* gene belongs to the K_n_ class of dehydrins, which respond to frost and drought, but they are not regulated by the light [37,39,52]. In the present experiment, we also observed that the *HvDHN5* gene did not respond differently at different light intensities. The lack of differences can also be explained by the fact that, although expressions of DHN family members have diurnal rhythms [53], temperature is also important (besides the photoperiod) to *BpuDhn1* and *BpuDhn2* genes to induce their expression in birch. Moreover, the short day length just helps to initiate the expression as the key component in this process [54]. It might suggest that the regulation of *HvDHN5* is independent from the phytochrome-mediated signalling pathway, which makes the barley cold acclimation mechanism more flexible. 

### 3.2. The Freezing Tests

The comparison was based on the white light treatments against the far-red modulated zones. (Figure 4). There was an unequivocal difference between the two treatments at every light intensity and temperature. It is known that light intensity itself affects the degree of frost tolerance, and by using adequately high light intensity the inherent frost tolerance can be enhanced even at room temperature in wheat [55]. Furthermore, without sufficient light during the cold hardening period, even winter cereals with a potentially high level of frost hardiness are incapable of achieving adequate freezing tolerance [25,56]. Similar findings were reported from *Arabidopsis*. In *Arabidopsis*, short photoperiods resulted in lower frost tolerance in plants, while low light intensity produced similar results. Although the experiment was not primarily intended to address this topic, the authors suggest that the development of frost tolerance in *Arabidopsis* is related to the total number of photons received from the emitting light, not to the duration of the photoperiod [57]. Our study (Figure 4) also confirmed these statements in barley plants as well, since from low to high intensity the relative conductance decreased at both temperatures, even without further far-red supplementation. In rye, it was reported that after cold exposure, rye plants showed much greater cold tolerance compared to their controls grown at normal temperature. In contrast, their results also showed that at higher light intensity, plants had elevated frost tolerance, but that increase was not as high as it was in the hardened controls [56]. In winter habit wheat (*Triticum aestivum* L. var. Mv Emese) similar results were proved. In cold hardened plants the frost tolerance of the seedlings was much higher when they were hardened at normal light intensity (250 PAR). Moreover, under high light intensity (500 PAR) the plants also showed greater survival rates than under low light conditions (20 PAR) even without cold hardening [58]. Our results showed this tendency as well, but our results also showed that the decrease in the R:FR ratio not only exhibited a synergistic effect with low temperatures [23], but it also triggered certain mechanisms at non-hardening temperatures, especially at high light intensity (350 PAR). In the case of decreased R:FR ratio, a greater freezing tolerance was observed at all light intensities. In tomatoes, the effects of additional far-red light during cultivation were studied after harvesting. The supplementary far-red light during cultivation improved postharvest cold tolerance in mature green and red harvested tomatoes as well. In this case, the supplementation caused higher firmness, faster color development, and also lowered chilling-induced pitting and decay in tomatoes [59]. Although the effects of complementary far-red light with high light intensity are beneficial in many cases, if the plants were previously acclimated to a lower incident light intensity in growth chambers, the sudden increase in light intensity can bring harmful side effects like photoinhibition or loss of photosynthetic and anthocyanin pigments [60,61] during cold acclimation. It has also been shown that the lower R:FR ratio can increase the level of freezing tolerance via influencing the phytochrome signalling system [23,24,48]. Our results also proved that the elevated FR light in the spectra can greatly improve the frost tolerance, which can be further improved by increasing the light intensity in barley. In woody plants, this effect of the reduced R:FR ratio was established long ago. For instance, in *Cornus* and *Weigela*, red light negatively influenced frost tolerance until far-red light was not added to the illumination. In another genotype of *Cornus*, the far-red light positively affected the cold acclimation process. [62,63]. In comparing the remarkable frost tolerance at high light intensity with the expression pattern of *COR14b*’s threshold (Figure 2), it may even suggest that the numerous mechanisms, which affect frost tolerance in barley [64], might be slightly redundant to each other, and they work together to determine the final response. 

## 4. Materials and Methods

### 4.1. Plant Materials and Growth Conditions

For this experiment a cold-tolerant, winter habit barley *Hordeum vulgare* spp. *vulgare* var. Nure was used. After three days of germination, seedlings were planted in Jiffy-7 peat pellets (Jiffy Group, Oslo, Norway). The plants were grown in a PGV-36 growth chamber (Conviron PGV36; Controlled Environments Ltd.; Winnipeg, MB, Canada) equipped with a modular LED light ceiling. In the developing phase plants were cultivated for 14 d at a constant 15 °C temperature, with 12 h photoperiods under only white light with a continuous wide-spectrum LED (Philips Lumileds, LXZ25790-y) at 250 photosynthetically active radiation (PAR) intensity (which is equal to µmol m^−2^·s^−1^).

### 4.2. Light Treatments

We divided the ceiling into 6 areas, and in three areas the light intensities were 125, 250, and 350 PAR (Figures S1–S3). We paired the rest of the areas, where we added supplementary FR illumination to the white light with a narrow 655 nm LED (Philips Lumileds, LXZ1-PA01). The red/far-red ratio was 0.5 above these plants. The temperature and the number of illuminated hours remained unchanged during the treatment, which lasted ten days. After 10 d, the temperature was lowered to 5 °C without any other changes in the zones. This phase was seven days long.

### 4.3. Gene Expression

On the first and last day of the treatments, 50 mg leaf samples were collected after 6 to 8 h of light. Total RNA was isolated using Direct-zol^TM^ RNA MiniPrepkit (Zymo Research Corp., Irvine, CA, USA) and was determined by a NanoDrop 2000 Spectrophotometer (Thermo Fisher Scientific Inc., Wilmington, MA, USA). The cDNA libraries were prepared with the Moloney Murine Leukemia Virus (M-MLV) Reverse Transcriptase and oligo (dT) 18 primer (Promega Corporation, Madison, WI, USA) according to the manufacturer’s protocol. The expression level of genes was revealed by CFX96 TouchTM real-time PCR Detection System (Bio-Rad Hungary Ltd., Budapest, Hungary) and KAPA SYBR^®^ FAST, Master Mix (2×), Universal qPCR Kit (Kapa Biosystems, Inc., Wilmington, MA, USA). The PCR primers were identical to previously published primers (Table S1; [65,66]). The relative expression levels were calculated by the ΔΔCt method [67]. Cyclophilin was used as the reference gene. 

### 4.4. The Freezing Tolerance of Leaf Segments

Samples were taken on the last day of each treatment to perform the freezing tests according to [68]. Then, 12 leaf segments (2 mm long) from four different plants were placed in Falcon tubes (Thermo Fisher Scientific Inc., Wilmington, MA, USA). Subsequently, the samples were placed in a GP200-R4 liquid freezing system (Grant Instruments, Shepreth, UK) where a 50% ethylene glycol solution was flowing continuously to ensure rapid heat transfer and uniform temperature. The temperature was continually reduced from treatment temperatures to −2 °C, where we kept the samples for 18 h to simulate cold acclimation as a field circumstance. Samples in the first treatment were frozen at −5, −7, and −9 °C, while the second treatment samples were frozen at −8, −10, and −12 °C for 1 h. After freezing, the samples were removed from the system, and MQ water was added to each tube. The samples were shaken for two hours before we measured the electrolyte leakage levels with a conductometer instrument (Mikro KKT, Budapest, Hungary). For the data analysis, Multi-Sample Conductometer version 1.0 (Intron Software, Biological Research Centre, Szeged, Hungary, (Copyright© L. Menczel, 2002)) was used. The relative conductance was calculated in five biological repetitions that originated from four different plants each.

## 5. Conclusions

According to our best knowledge, these are the first results to show that the combined effects of cold, light intensity, and modification of the R:FR light ratio can greatly influence the expression pattern of *HvCBF14* and *HvCOR14b* genes and can also adjust the level of the *HvDHN5* gene. Interestingly, the two genes, which are used primarily as markers to predict the level of barley frost tolerance, behaved differently. While the *HvCOR14b* gene showed an absolute increase in both low-temperature and under supplemented far-red light conditions, *HvDHN5* only responded to low temperatures in a reliable manner. This suggests that the *HvDHN5* gene should be used when light modifications do not occur or to predict the level of total hardiness after adequately long cold acclimation. Therefore, we suggest that in those cases, when artificial light sources are used during cold hardening, the expression level of *HvCOR14b* should be monitored instead. These three environmental factors seem to be interfering with each other during the cold acclimation process, which can result in serious alterations in the level of frost hardiness. Accordingly, during selection for frost tolerance in plant growth chambers, it is very important to keep both the light intensity and spectrum constant, and this is the prerequisite for repeatability. Moreover, in field conditions applying external light sources, it also might prevent cold injuries in different plant species during autumn. Of course, this hypothesis must be proved experimentally. We are currently investigating the hormone signalling and lipidomics behind this phenomenon since this work strongly proposes a deeper investigation for a better understanding of the whole process. 

## Supplementary Materials

The following are available online at https://www.mdpi.com/2223-7747/9/1/83/s1, Figure S1: Spectral composition of modulated light treatments at 125 μmol m^−2^·s^−1^ intensity, Figure S2: Spectral composition of modulated light treatments at 250 μmol m^−2^·s^−1^ intensity Figure S3: Spectral composition of modulated light treatments at 350 μmol m^−2^·s^−1^ intensity. Table S1: Primer sequences and references.
