# Supplementary material for: Temperature and Light-Quality-Dependent Regulation of Freezing Tolerance in Barley
Source: Plants (Basel). 2020 Jan 9;9(1):83. doi: 10.3390/plants9010083 (PMC7020399; doi:10.3390/plants9010083)
Supplement: Supplementary file 1 [file plants-09-00083-s001.pdf]

plants

Temperature and Light-quality Dependent Regulation of Freezing Tolerance in Barley

Mohamed Ahres, Krisztián Gierczik, Ákos Boldizsár, Pavel Vítámvás and Gábor Galiba

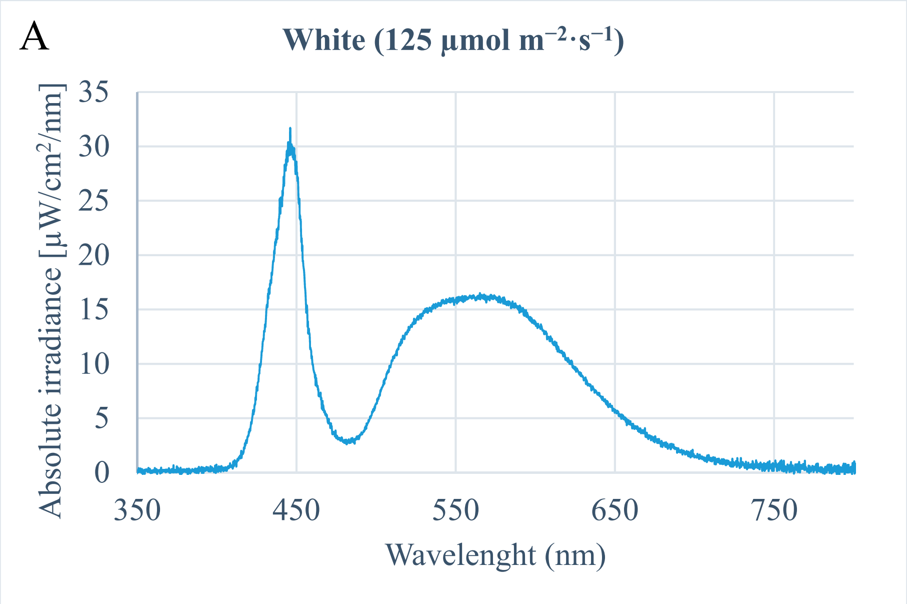

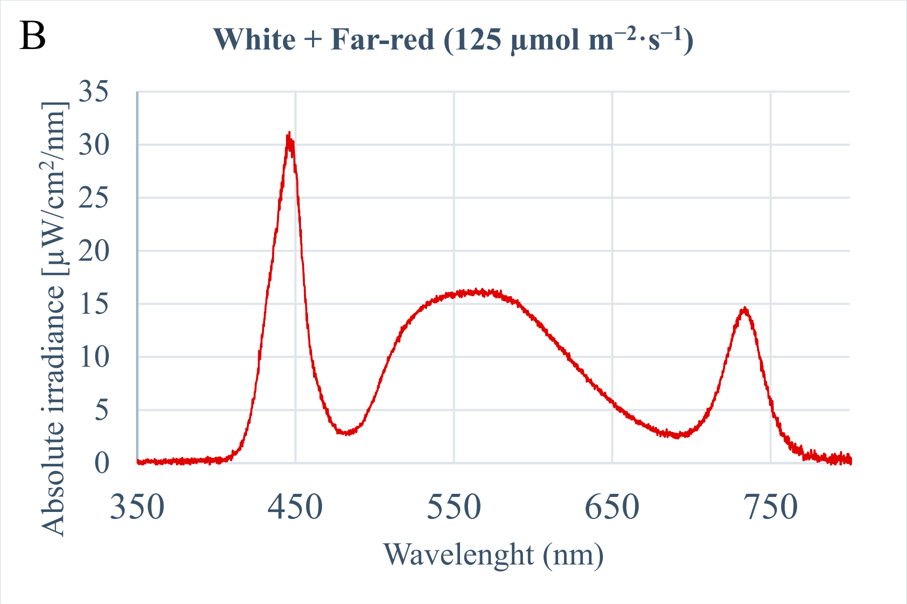

Figure S1. Spectral composition of modulated light treatments at 125 μmol $m^{-2}{\cdot}s^{-1}$ intensity. (**A**) the spectral composition of the white light, (**B**) the spectral composition of white light supplemented by extra far-red light.

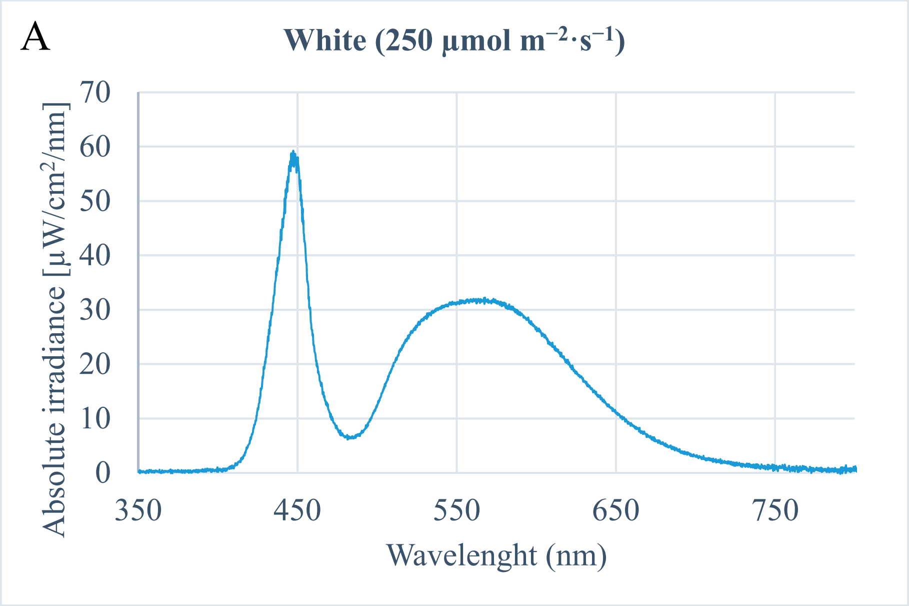

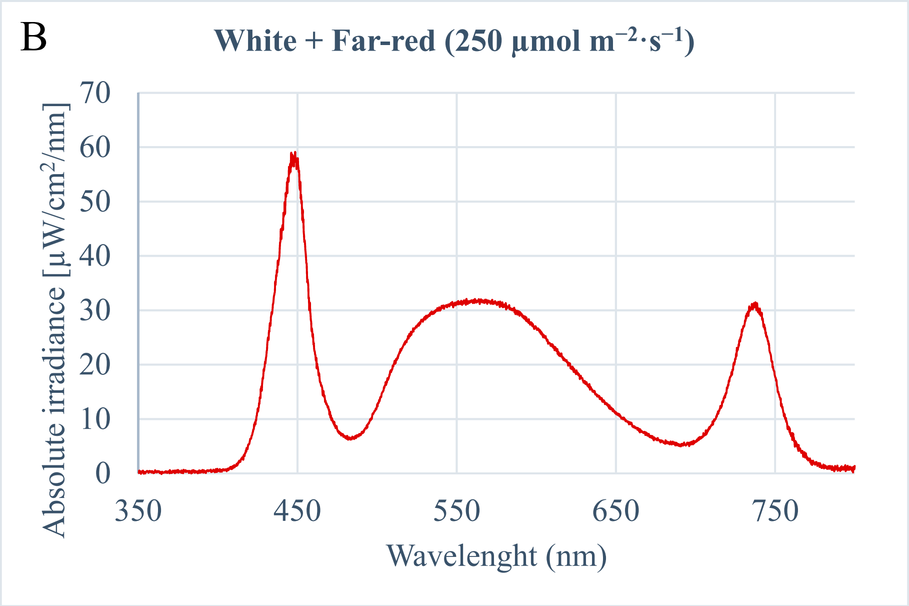

Figure S2. Spectral composition of modulated light treatments at 250 μmol $m^{-2}{\cdot}s^{-1}$ intensity. (**A**) the spectral composition of the white light, (**B**) the spectral composition of white light supplemented by extra far-red light.

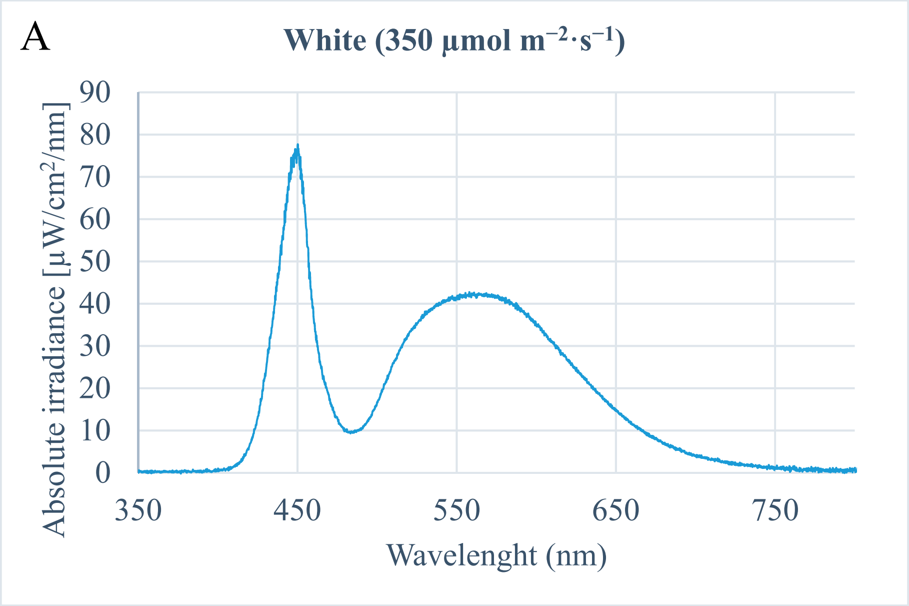

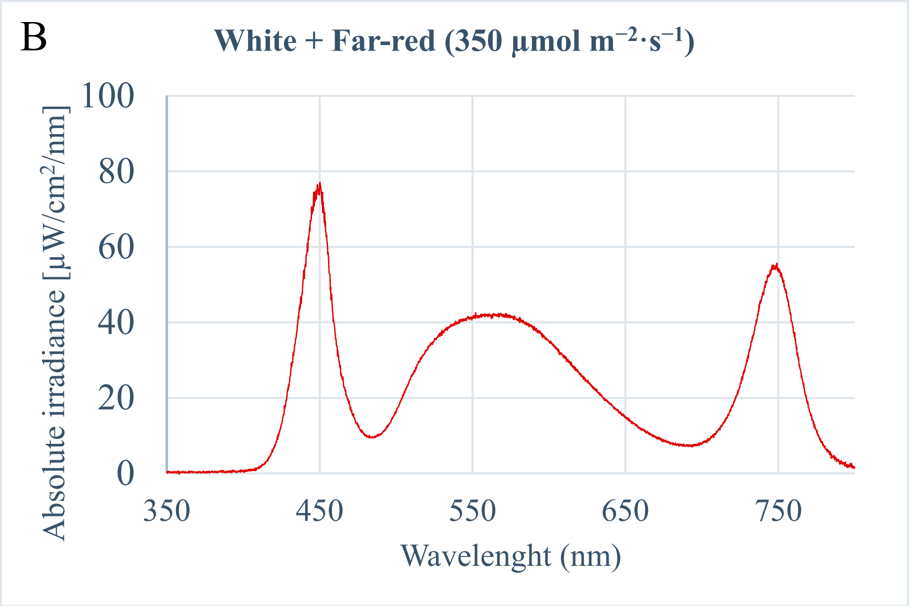

Figure S3. Spectral composition of modulated light treatments at 350 µmol $m^{-2}{\cdot}s^{-1}$ intensity. (**A**) the spectral composition of the white light, (**B**) the spectral composition of white light supplemented by extra far-red light.

Table S1. Primer sequences and references.

**Primer Sequences and References**			
**Name of the Gene**	**Forward Sequences 5'-3'**	**Reverse Sequences 5'-3'**	Reference
Cyclophilin	CCTGTCGTGTCGTCGGTCTAAA	ACGCAGATCCAGCAGCCTAAAG	Burton et al., 2004
HvCBF14	GTTGAATGAGCACTGGTTTGG	ACAATGAACGAGCAGGTATGG	Morran et al., 2011
HvCor14b	TTGAGGATGTGAGCAAATGAG	TACATCGTCAATGACGAGACC	
HvDHN5	TGGCGAAGTTCCACCGTATGC	ACGAAAACTGTTGCCACACTG	
